# Undergraduate teaching of surgical skills in the UK: systematic review

**DOI:** 10.1093/bjsopen/zrad083

**Published:** 2023-10-11

**Authors:** Sean C Glossop, Hari Bhachoo, Thomas M Murray, Rayan A Cherif, John Y Helo, Evie Morgan, Arwel T Poacher

**Affiliations:** School of Medicine, Cardiff University, Cardiff, South Glamorgan, UK; School of Medicine, Cardiff University, Cardiff, South Glamorgan, UK; School of Medicine, Cardiff University, Cardiff, South Glamorgan, UK; School of Medicine, Cardiff University, Cardiff, South Glamorgan, UK; School of Medicine, Cardiff University, Cardiff, South Glamorgan, UK; School of Medicine, University of St Andrews, St Andrews, Fife, UK; Department of Plastic Surgery, St Thomas’ Hospital, Guys and St Thomas’ Trust, London, UK

## Abstract

**Background:**

Students must be proficient in surgical skills according to General Medical Council and Royal College of Surgeons of England guidelines. If these skills are not appropriately taught, there is a risk of an incoming junior workforce with inadequate surgical skills. This paper aimed to review the literature relating to undergraduate teaching of surgical skills in the UK and summarize future suggested training methods.

**Methods:**

The databases MEDLINE, Embase and SCOPUS were searched, and the existing literature relating to methodology of undergraduate teaching of surgical skills in the UK over the past 10 years was summarized. The Medical Education Research Quality Instrument was used to assess research quality.

**Results:**

A total of 19 papers were included. Cross-sectional evaluations and survey-based studies highlight a clear deficit in surgical skills teaching in the UK. Medical students are currently unable to fulfil their own learning needs and meet requirements set out by the General Medical Council. This lack of surgical teaching appears to negatively affect student desire to pursue a surgical career. The three main themes for improvement are extracurricular surgical skills days, near-peer teaching and simulation. Each method appeared to improve learning, although no studies utilized medium- to long-term follow-up to demonstrate efficacy and there lacks a clear consensus as to the ‘standard’ of undergraduate surgical skill education. There was also potential for selection bias and response shift bias in many of the studies assessing pre- and postintervention confidence and opinions.

**Conclusion:**

There is a concerning lack of surgical skills teaching that has resulted in medical students and junior doctors not having the necessary surgical skills as per General Medical Council guidance and students feel that their own learning needs are not met. This failure to address the learning deficit may be responsible for the fall in surgical competition ratios. While surgical skills teaching must be improved urgently, more robust evidence is required to evaluate the optimal ways of approaching this issue.

## Teaser

Undergraduate medical students must be proficient in certain surgical skills as set out by General Medical Council and Royal College regulations. There appears a deficit in such teaching which we have discussed and attempted to address in terms of alternative methodology reported in the literature that can be used to teach these basic surgical skills.

## Introduction

Surgical skills teaching is an important aspect of the undergraduate medical curriculum in the UK. The General Medical Council (GMC), an independent regulator for doctors practising in the UK, has outlined the required surgical skills a medical graduate must show proficiency in to be deemed a competent and safe healthcare professional. These skills are surgical scrubbing, wound closure and the use of local anaesthetics, which appear in the GMC’s ‘Tomorrow’s Doctors’^1^ guidance and the more recent ‘Outcomes for Graduates’ practical skills guidance^2^.

The Royal College of Surgeons of England (RCSE) also stipulates guidance for undergraduate curriculum learning, listing the skills in line with the GMC guidance, as well as 35 surgical conditions that undergraduates should be able to recognize^3^. However, there is little evidence evaluating the quality of delivery of basic surgical skills for medical students in the UK. Furthermore, any deficits in training could potentially have an impact on preparedness and required basic knowledge after qualifying in an undergraduate medical degree. This poses not only a danger to patients but discourages students from pursuing a career in surgery^4,5^, important in the context of declining national competition ratios^6^. However, due to variances in teaching methods across British medical schools, these discrepancies are poorly documented in the current literature, with the last review almost a decade ago reporting a severe deficit in the provision of surgical skills teaching in the undergraduate medical curriculum^7^.

Therefore, the aim of this study was to demonstrate the current state of undergraduate surgical skills teaching in the UK, and to evaluate alternative approaches or interventions that have been used to teach medical students and whether these methods have been successful. This will enable an informed discussion relating to any potential reported deficits, as well as alternative methods that may be useful to improve and strengthen curricula within medical schools across the UK.

## Methods

This review was prospectively registered with the International Prospective Register of Systematic Reviews (PROSPERO) (CRD42023389990).

Throughout the development and production of this systematic review, the PRISMA guidelines^8^ were followed.

MEDLINE, Embase and SCOPUS databases were searched for suitable literature. A manual search of Google Scholar was also undertaken. The database search terms were (‘medical student’ OR ‘student’ OR ‘undergraduate’) AND (‘surgical skills’ OR ‘basic surgical skills’ OR ‘suturing’ OR ‘knot tying’ OR ‘local anaesthetic’) AND (‘prepared*’ OR ‘confidence’ OR ‘knowledge’ OR ‘approach’ OR ‘teaching method’) AND (‘United Kingdom’ OR ‘UK’).

Database search limits were set to articles written in English between 2012 and 2022 to maintain the relevance and currency of this review. Pandemic years were included to capture papers relating to alternative teaching methods that were brought on through necessity during the coronavirus (COVID-19) pandemic. Ethical approval was not required for this systematic review.

### Inclusion and exclusion criteria

Articles were considered for inclusion in the review if they met the agreed criteria (*Table 1*). Articles that related to surgical skill teaching amongst medical students on the undergraduate medical degree in the UK were included. The purpose of this review was to establish literature relating to a deficit of surgical skill teaching in undergraduate medicine and determine any related articles reviewing alternative methods or approaches to such teaching.

**Table 1:** Inclusion and exclusion criteria

Criteria	Inclusion	Exclusion
Research participant	Studies discussing undergraduate teaching of surgical skills	Research relating to a research participant other than surgical skills teaching in the UK
Participants	Studies involving students who are receiving their undergraduate medical teaching at a UK medical school or studies surveying medical schools	Any studies from non-UK medical schools and studies that focus only on postgraduate teaching
Location	UK only	Any studies from outside the UK
Type of studies	Original and primary research	Commentaries, abstracts, conference abstracts
Methodology	Quantitative/qualitative/mixed methodology	
Timescale	Literature published between 2012 and 2022 (last 10 years)	Literature published before 2012

### Title and abstract review

A three-tier reviewer method comprising two reviewers per panel was used to reduce the risk of bias. After duplicate papers were excluded, abstracts and titles of each article were read independently by two authors (S.G., H.B.). A shared Microsoft® Excel (version 2303, Microsoft, Redmond, WA, USA) spreadsheet was created to document articles that met the criteria of inclusion in preparation for reviewing full texts. Any discrepancies regarding abstract and title screening were discussed to reach an agreement. If consensus on eligibility for any abstracts could not be reached, they were passed to a second panel of two authors (T.M., R.C.) who independently analysed the abstracts to assess whether they met the criteria of inclusion and to reach a decision.

### Full-text review and data extraction

**Fig. 1:**
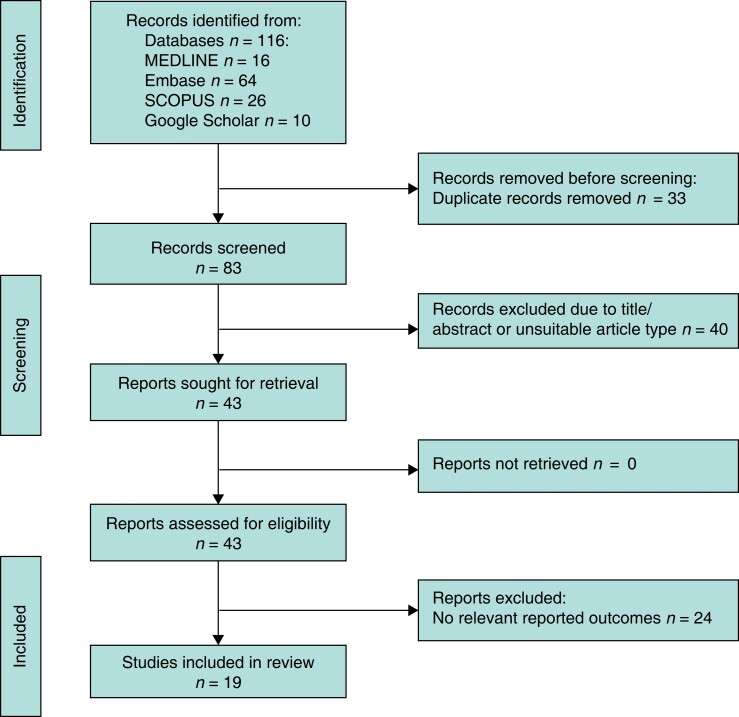
PRISMA flow diagram demonstrating the literature search and study selection process

Full texts of each article were independently screened by two authors (T.M., R.C.). For any disagreements regarding articles and for which a consensus could not be reached, a third panel of authors (J.H. and A.P.) read the article and decided whether it met the criteria; articles decided upon following this process were then placed in a spreadsheet. *Figure 1* shows the PRISMA flow chart of the methods of inclusion and exclusion^8^.

The quality of articles was scored by two authors (S.G., H.B.) with any disagreements being assessed by the senior author (A.P.). The Medical Education Research Quality Instrument (MERSQI)^9^ was used and appraises quality based on six domains (study design, sampling, type of data, validity of evaluation instrument, data analysis, outcomes), each scoring a maximum of 3 points. The possible score range of using this tool is 5–18, as five domains have a minimum score of 1, with a score of 18 indicating the highest quality. This tool was chosen after having registered the protocol, due to heterogeneity amongst study methods in the literature and the mixed-methods approach to this review. Articles were not excluded based on this score; however, this system enabled an informed assessment of bias and research quality in each article, allowing a narrative synthesis discussion of reliability.

## Results

Nineteen studies were included in this review and were assessed within the domains of evaluation of current undergraduate training standards, methodology of improving surgical skills teaching and the influence of undergraduate practical teaching on career choice.

### Quality assessment

Eighteen of the included articles were suitable for assessment using the MERSQI score, as one study involved qualitative data. Scores ranged from 8.5 to 14.5, with a mean(s.d.) of 10.8(1.4). (*Table 2*).

**Table 2:** Total MERSQI scores for 18 of the included studies

Study	MERSQI score
Lee *et al*. (2016)^10^	10
Davis *et al*. (2014)^7^	10.5
Rufai *et al*. (2016)^11^	10.5
Lee *et al*. (2020)^12^	8.5
Hakim *et al*. (2019)^13^	9
Kuo *et al*. (2022)^14^	11
Spiers *et al*. (2018)^15^	11
Spiers *et al*. (2019)^16^	12
George *et al*. (2017)^17^	11.5
Mughal *et al*. (2015)^18^	12
Down *et al*. (2020)^19^	11
Chidambaram *et al*. (2019)^20^	14.5
Bennett *et al*. (2018)^21^	11
Preece *et al*. (2015)^22^	11
Sutton *et al*. (2014)^23^	10.5
Rouhani *et al*. (2017)^24^	9.5
Ologunde *et al*. (2015)^25^	9
Hamaoui *et al*. (2013)^26^	11

MERSQI, Medical Education Research Study Quality Instrument.

### Current undergraduate training standards

A cross-sectional study by Lee *et al.*^10^ received 328 responses from students across 31 medical schools in the UK. Only 65 per cent of the students felt prepared for their surgical placement, significantly less than the 88 per cent who felt prepared for their medical placements. This may be influenced secondary to only 45 per cent of students who felt confident in their suturing ability and 60 per cent of students who felt confident in the infiltration of local anaesthetic. However, only 33 per cent of students found their undergraduate surgical science teaching in medical school to be ‘adequate’. Additionally, only one medical school ran an adapted version of the basic surgical skills course developed by the RCSE. The study concluded that it was necessary to review ‘undergraduate surgical provision’ to ensure that students can practise safely for foundation jobs in surgery. The results of this are reinforced by those of Davis *et al.*^7^ who conducted a national review of surgical skills training in medical schools across the UK. Twenty-three medical schools responded to a survey which showed that ‘gowning and gloving’ was taught by 72.8 per cent of the schools, while ‘handling instruments’ was taught by just 29.4 per cent, ‘suturing techniques’ by 24.7 per cent and ‘knot tying’ by just 17.4 per cent of medical schools.

Likewise, another study by Rufai *et al.*^11^ examined the responses of 705 students from 16 different medical schools across the UK. Eighty-six per cent (607/705) of these students reported being taught about suturing in medical school in line with the curricula, but just 13.5 per cent (82/607) of these students thought that the training provided through the curriculum was adequate for suturing skills. These national studies provide useful clarity on the current surgical skills teaching provision, suggesting a more systemic issue with undergraduate surgical teaching. Therefore, the literature demonstrates a clear and consistent deficit of surgical skills teaching in the UK, evidenced by the poor perceptions of medical students relating to both their surgical skill teaching and ability.

### Methods of teaching surgical skills

#### The surgical skills day

There have been numerous attempts to address the deficit in undergraduate surgical skills teaching (*Table 3*), one of the most popular being ‘surgical skills day’ (SSD), which is a practical course involving the teaching of various surgical skills run over the course of a day. Lee *et al*.^12^ conducted a study involving an SSD hosted by a surgical society at Bristol Medical School. Twenty-nine students were assessed on suturing skills before and after instruction by UK surgeons using a binary system (0 for incompetency and 1 for competency), which showed a mean(s.d.) improvement of 3.92(0.40) points (*P* < 0.001). This study concluded that such training events should be incorporated into curricula across medical schools to ‘address skills deficits in current undergraduate programmes’.

**Table 3:** Impact of various surgical skill teaching methods and their assessment on undergraduate learning

Study	Sample	Measure of intervention	Preintervention score	Postintervention score	Significance
**Surgical skills day**					
Lee *et al*. (2020)^12^	29 medical students	Assessment of suturing skills by UK surgeons at various stages of training	Mean competency score 1.8(0.33)	Mean competency score 5.72(0.17)	*P* < 0.001
Kuo *et al*. (2022)^14^	58 medical students	Impact of skills day on the perceived confidence in performing skill and preparedness for future practice	Not disclosed	Not disclosed	Significant increase in confidence in performing all GMC recommended surgical skills (*P* = 0.0001), preparedness for clinical placements (*P* = 0.0001) and work as a junior doctor (*P* = 0.01)
Spiers *et al*. (2018)^15^	27 medical students	Impact of perceived confidence in performing basic plastic surgical skills	Suturing: 2.68/5, tying knots: 2.32/5, local flap transposition: 2.14/5, importance of (flap) geometry: 2.55/5, tendon repair: 2.05/5	Suturing: 4.00/5, tying knots: 3.68/5, local flap transposition: 3.82/5, importance of (flap) geometry: 4.14/5, tendon repair: 4.05/5	Not disclosed
Spiers *et al*. (2019)^16^	36 medical students	Confidence in performing tracheostomies	1.26/5	4.43/5	*P* < 0.0001
**Simulation for learning**					
Mughal *et al*. (2015)^18^	66 medical students across 6 UK medical schools	Confidence, self-perceived competence and knowledge before, immediately following and 8 weeks after the workshop	Mean confidence in assessing an unwell surgical patient: 2.5/5, mean confidence in commencing initial management: 2.7/5, MCQ mean score: 57.9%, assessing the acute surgical patient: 2.5/5, ordering investigations: 2.9/5, initiating management: 2.7/5	Mean confidence in assessing an unwell surgical patient: 4.4/5, mean confidence in commencing initial management: 4.1/5, MCQ mean score: 70.9%, MCQ mean score at 8 weeks postcourse: 69%, assessing the acute surgical patient: 4.4/5, ordering investigations: 4.2/5, initiating management: 4.1/5	MCQ mean score: *P* < 0.0001 MCQ mean score at 8 weeks postcourse: *P* = 0.0039 All 12 tested surgical domains: *P* < 0.0001
Down *et al*. (2020)^19^	14 medical students	Perceived confidence in performing surgical skills	Not disclosed	Handling instruments +0.65, interrupted suturing +0.9, continuous suturing +1.45, subcuticular suturing +1.33, knot tying +1.22, excision and closure +1.59, bowel anastomosis +2.28, tendon repair +1.78, artery ligation +1.33	Handling instruments *P* < 0.001, interrupted suturing *P* < 0.001, continuous suturing *P* < 0.001, subcuticular suturing *P* < 0.0001, knot tying *P* < 0.05, excision and closure *P* < 0.0001, bowel anastomosis *P* < 0.0001, tendon repair *P* < 0.01, artery ligation *P* < 0.05
Chidambaram *et al*. (2019)^20^	21 medical students in the intervention group and 17 medical students in the control group	Comparison of written information and simulation training for performing surgical procedures	Mean cognitive performance score, control group: 24.7	Mean cognitive performance score, intervention group: 41.9	Not significant *P* > 0.05
**Near-peer learning**					
Bennett *et al*. (2018)^21^	70 medical students	Confidence levels in performing 8 surgical domains	Not disclosed	WHO surgical safety checklist: +3.94, scrubbing: +2.99, gowning/gloving: +3.34, knot tying: +5.53, interrupted sutures: +5.89, continuous sutures: +6.53, vertical mattress sutures: +6.46, local anaesthesia: +3.73	*P* < 0.05 for knot tying, interrupted sutures, continuous sutures and vertical mattress suture
Preece *et al*. (2015)^22^	35 medical students	Number of sutures completed	2.3(1.6)	5.3(1.7)	*P* < 0.001

Values are mean(s.d.) unless otherwise indicated. GMC, General Medical Council; MCQ, multiple choice question.

The use of a single day of surgical skills has been further evaluated by Hakim *et al.* and Kuo *et al.*^13,14^.. Hakim *et al.*^13^ evaluated the use of an annual SSD and found that 79 per cent of students felt that the SSD made them more likely to pursue a career in surgery. In this workshop, students were taught a variety of skills using eight stations, including suturing and more complex surgical skills, and for all stations, the reported satisfaction of students was above 7 on a scale of 1–10 (10 as the highest). Kuo *et al.*^14^ demonstrated that in-person small group teaching to 58 phase-one medical students (years 1 and 2) by qualified junior doctors greatly improved confidence levels for all GMC-mandated surgical skills (*P* < 0.0001). Therefore, the benefits of single-day early exposure to surgical skills for undergraduates could potentially improve surgical skill abilities amongst cohorts.

Spiers *et al.* have conducted a number of one-day specialist surgery courses through their surgical societies^15,16^. In their 2018 study relating to plastic surgery^15^, trained surgeons instructed students in basic knot tying, suturing and more advanced skills related to the specialty such as tendon repair. All skills saw increases in self-reported confidence levels. In their 2019 study on augmentation of ear, nose and throat (ENT)surgery using a similar model^16^, they saw a statistically significant increase in confidence levels for tracheostomy (*P* < 0.0001) which is a more advanced procedure not currently taught as part of medical curricula. Furthermore, George *et al.*^17^ replicated these results in undergraduate students following interactive cardiothoracic workshops involving numerous skills, including the dissection of animal hearts as well as suturing and knot tying. Eighty responses to Likert-style questions were analysed and it was found that average Likert scores increased after the workshop (*P* = 0.001) compared with pre-workshop averages, which represented a 28 per cent increase in interest in cardiothoracic surgery. Whilst the relatively small number of participants in the study as well as the style of questioning may be limiting, it shows the potential of focused and more niche surgical skill workshops which can increase interest in specific surgical specialties whilst concurrently involving the teaching of basic surgical skills.

However, none of these studies demonstrated or even attempted to evaluate sustained improvement in surgical skill or improvement in confidence level over a long interval. This is a key marker of the efficacy of an intervention and the adequacy of a single day to address the deficiency in surgical skills among the UK's undergraduate medical students and is not documented in the literature.

#### Simulation for learning

Simulation has been well investigated within the postgraduate curriculum^27^, however, this is less well covered in the context of undergraduate learning. A study by Mughal *et al.*^18^ provides an evaluation of a simulation workshop that taught surgical skills. A rating scale questionnaire given to participants comprising eight confidence statements and four self-competence statements demonstrated significant improvement after the intervention (*P* < 0.0001) compared to before. Additionally, it was found that all students (*n* = 56) thought that the medical curriculum should include more simulations, with 58.6 per cent strongly agreeing, suggesting simulation as a useful mechanism of surgical skills education.

A study by Down *et al.*^19^ designed teaching sessions using a constructivist approach to teach a small group of penultimate-year medical students based on GMC-required outcomes as well as some advanced surgical skills. This involved introducing the group to the basic skills and then providing more advanced tasks related to clinical scenarios. They compared the data before and after the session which showed statistically significant improvements in mean confidence levels for all the basic skills, as well as advanced skills (*P* < 0.001 for instrument handling, interrupted suturing and continuous suturing; *P* < 0.05 for knot tying; *P* < 0.0001 for subcuticular suturing). This study gives a platform for the exploration of more structured approaches to teaching surgical skills and is thus useful to the study aims, despite having been conducted on a very small group.

In a more focused study, Chidambaram *et al.*^20^ conducted a randomized controlled trial comparing a group of 40 students who were either allocated a control group, which comprised written instructions for students, or an intervention group, in this case, touch surgery training. Touch surgery involves the use of an interactive mobile application available on smart devices. It was found that for the touch surgery group, mean performance was better than the control group although this was not statistically significant. While this study focuses on the more advanced application of surgical skills, it gives another possibility for improving surgical learning standards amongst medical students using technology and simulation.

#### Near-peer learning of practical skills

Comparably, another study by Bennett *et al*.^21^ also underlines the potential of medical students learning from their peers. They organized five courses delivered by senior medical students to 70 junior students covering the WHO surgical safety checklist, surgical scrubbing, gowning and gloving, knot tying, interrupted suturing, continuous suturing, vertical mattress sutures and local anaesthesia. There was a statistically significant increase in the confidence levels following the course (*P* < 0.05 for knot tying, interrupted sutures, continuous sutures and vertical mattress sutures) although increases were seen in mean scores for all of the eight skills taught. Additionally, 80 per cent (*n* = 56) agreed that their desire to pursue a career in surgery was strengthened by the peer-assisted learning course. Preece *et al.*^22^ similarly showed that peer-assisted learning can be beneficial to teach basic surgical skills. Thirty-five students from a British medical school attended an event led by senior medical students in which they were taught basic suturing. Students were assessed before and after being taught by peer tutors. The study found that the number of sutures that students were able to complete increased from 2.3(1.6) pre-teaching to 5.3(1.7), which was statistically significant (*P* < 0.001). This supports that peer teaching of basic surgical skills can improve outcomes for medical students when delivered in addition to the teaching they experience in their undergraduate degree.

Furthermore, Saleh *et al*.^28^ talk about a ‘peer-assisted learning’ experience in which a participant, tutor and session organizer qualitatively recount their thoughts about a specific near-peer session for basic surgical skills teaching. The session organizer reported that it was a ‘challenging experience’ although it helped them to improve their ‘organizational and leadership skills’. The peer tutor explained that the preparation for the sessions and then assisting in them ‘positively reinforced my own abilities’ and helped to form ‘strengthened relationships’ with peers. From the preclinical student’s perspective, they were ‘initially apprehensive about how useful the session would be’ although went on to explain that due to the ‘informal environment and ample time allotted’ they were able to concentrate on being taught the various skills and felt the advice given in the session helped to ‘allay my anxiety and smooth my transition into the clinical environment’. Whilst this paper is limited in structure, it does highlight the fact that peer-assisted learning can be beneficial and gives us an insight into student opinion, essential for developing an improved basic surgical skill teaching curriculum.

#### Influence of undergraduate practical teaching on career choice

The quality of undergraduate teaching may be a deciding factor in whether medical students pursue a surgical career. Sutton *et al.*^23^ collected data from 482 students from 20 medical schools through a survey. Only 71 per cent of students were able to have access to surgical skill training in medical school and it was found that the most common two reasons discouraging medical students from pursuing a career in surgery were experiences in terms of surgical exposure on clinical placement during the degree (43 per cent) and work experience in surgery (35 per cent). This study therefore demonstrates that exposure to surgery and experience in the field or lack thereof may influence medical students in terms of the career or specialty that they wish to pursue.

Another survey by Rouhani *et al.*^24^ received responses from 137 students from 20 different medical schools. When asked about surgical skills, 110 students responded, with over half (*n* = 56) agreeing that the provision of basic surgical skill teaching made them ‘more likely to pursue a career in surgery’. This study highlights the importance of basic surgical skills in undergraduate medical education not only in ensuring institutions are producing competent graduates but reigniting the desire of graduates wanting to pursue a surgical career.

The introduction of surgical workshops is additionally welcomed by medical students, particularly with regard to career paths. Ologunde *et al*.^25^ received 60 responses to a cross-sectional survey in which students were asked about the ‘most useful career-guiding events’ provided by the societies. Of the 10 available categories, the most popular was ‘surgical skills workshops’ in which over a fifth (21.9 per cent) of respondents believed that these workshops were the most beneficial in helping to decide careers. While being part of such societies is optional in undergraduate medicine, this study underlines the fact that surgical workshops can shape the perspectives of medical students on future career paths. Hamaoui *et al.*^26^ designed a programme in which 47 medical students at a British university experienced teaching four key surgical skills over the course of 4 weeks. This was in accordance with the RCSE's guidelines. Using Likert pre- and postcourse questionnaires to gather data in terms of the students’ perceptions, they found a statistically significant increase (*P* < 0.01) in student confidence levels for the teaching of surgical skills. Additionally, it was found that 70 per cent of the students agreed that attending such a course could be influential in pursuing a surgical career.

*Table S1* provides a full breakdown of all 19 papers included in this review section.

## Discussion

The literature demonstrates a concerning deficit in surgical skills training with students consistently failing to meet GMC requirements of minimum basic surgical skills in the undergraduate curriculum. Furthermore, the absence of effective teaching of surgical skills deters students from considering a career in surgery. This is deeply concerning, considering the declining specialty application competition ratios, with general surgery competitiveness falling dramatically from 1:15.0 in 2009 to 1:2.16 in 2019^29,30^ compounded with burnout amongst surgical trainees^31^.

The cross-sectional survey evaluations of medical students over the last decade have demonstrated inadequate coverage of the RCSE's surgical curriculum^3^ and of the key GMC requirements^2^. Furthermore, only 30 per cent of students felt that their teaching was appropriate and safe and only 10–15 per cent felt prepared for their foundation training with the current undergraduate curriculum^7,10,11.^ This deficit has commendably, but concerningly, been partially filled by local student-led societies providing events for interested students. However, these day-long one-off, occasionally annual events^12–16^ were only available to a small number of students and rely on the willingness of proactive students to give up their time outside of the core curriculum. Furthermore, there were no studies that demonstrated a sustained improvement in surgical skills at follow-up, which has been shown to be an important marker of educational efficacy^32,33^. Clearly, this is not an appropriate way to address the deficit as it only benefits a small number of students and has not demonstrated long-term improvement. However, this methodology of covering a large volume of surgical skills in a single-day course should be evaluated with long-term follow-up and could then provide a realistic, resource-light methodology for addressing this deficit in surgical education.

The use of peer-assisted learning may be another way for medical schools to provide resource-efficient teaching to address the educational deficit in the short term. All the studies reporting on near-peer learning demonstrated a significant short-term increase in career aspirations and surgical ability^21,22,28^. Furthermore, this would not require the same number of clinical staff to make time in an already highly pressured postpandemic system, but instead, make use of senior medical students and junior doctors to provide a foundational understanding of these surgical skills. Through these sessions, senior students would also be able to develop their understanding of being a surgical teacher and build mentorship abilities that improve career performance^34^. However, there should be an assessment of the viability and of the medium- to long-term improvement of these surgical skills, with a direct comparison to senior-led teaching to ensure that the practice is safe and effective for learning^35^.

Simulation is poorly reported upon in the literature in relation to an undergraduate cohort whose learning needs are far simpler than those of surgical trainees. Whilst the impact of simulation has been well documented to provide benefit to postgraduates^27,36,37^, there is limited evidence to demonstrate the efficacy of simulation training for the undergraduate. Whilst the literature only exhibits short-term improvements in satisfaction amongst students and does not report on long-term skill-based outcomes, it gives us a basis for improvement of the current undergraduate teaching standards. However, at the postgraduate level, surgical boot camps for core surgical trainees have been shown to improve long-term success rates for candidates applying for higher surgical training^38,39^. A similar model could have potential translational benefits for skills training and further research would be useful to evaluate this model in the undergraduate setting.

Currently, surgical skills teaching is left to individual medical schools to develop in line with GMC guidelines. However, it appears that surgical societies bear much of the burden of extracurricular teaching for surgical skills. This is inappropriate given that teaching such skills requires multiple attempts at practice and training and should therefore be provided at multiple compulsory points of the undergraduate degree to better prepare students for their role as a doctor. These newly qualified doctors will be sent onto the wards with the assumption that they have fulfilled their GMC competencies, and will be expected to be able to suture a wound, scrub into theatre and apply local anaesthetic safely. These are the competencies of a safe practitioner, not just a surgeon. It appears that this deficit may also exist on an international scale, with the literature describing surgical education as inadequate on a global scale in both more and less economically developed countries^40–42^.

The studies included in this review had small populations which limited their power. Additionally, the majority of pre- and postintervention surveys reflected short-term outcomes of the teaching methods assessed and thus it may not reflect on the long-term impact of such techniques on learning. Certain studies have utilized parametric assessment methodology intended for non-parametric data that has overestimated the statistical evaluation of their impact. Whilst this does not affect how the raw data can be interpreted, it raises questions regarding study conclusions and future studies should provide a robust, transparent and appropriate statistical evaluation.

Furthermore, not only were the majority of these studies limited by obvious methodological flaws, but there was also little to no adjustment or even consideration for the sources of bias, particularly selection/participant bias and data collection. To mitigate the risk of selection bias, studies should ideally evaluate randomized, blinded cohorts. However, when this is not possible secondary to ethical or resource limitations, the Checklist for Reporting Results of Internet E-Surveys (CHERRIES) questionnaire guidance^43^ should be utilized in an attempt to mitigate this bias. Additionally, assessment of improvement in skill should assess not just student confidence, a relatively poor marker of ability^44^, but instead should utilize independent assessment by ‘trained’ trainers. Finally, correlation with the performance through self-reflection and supervisor-based assessment in the clinical context would also be a useful measure to reflect transferable improvements in ability.

The current undergraduate teaching of surgical skills in the UK is inadequate, indicating the potential presence of a significant number of UK doctors without the adequate surgical skills training required to be competent and safe professionals. Developing surgical workshops, small group learning and peer-assisted learning in line with current undergraduate guidance appears able to address the current deficit in undergraduate curricula. However, further evaluation is required to provide a ‘standard’ of undergraduate surgical education as well as to assess the long-term effectiveness of teaching methods. Additionally, where possible, it would be beneficial to relate this surgical skills teaching to clinical outcomes.

## Supplementary Material

zrad083_Supplementary_Data

## Data Availability

The authors confirm that the data supporting the findings of this study are available within the article and its *Supplementary materials*.
