# Supplementary material for: Undergraduate teaching of surgical skills in the UK: systematic review
Source: BJS Open. 2023 Oct 11;7(5):zrad083. doi: 10.1093/bjsopen/zrad083 (PMC10566575; doi:10.1093/bjsopen/zrad083)
Supplement: zrad083_Supplementary_Data [file zrad083_supplementary_data.docx]

**Undergraduate teaching of surgical skills in the United Kingdom: a systematic literature review**

Sean C. Glossop^1^, Hari Bhachoo^1^, Thomas M. Murray^1^, Rayan A. Cherif^1^, John Y. Helo^1^, Evie Morgan^2^, Arwel T. Poacher^3^

^1^Cardiff University, School of Medicine, Wales

^2^University of St Andrews, School of Medicine, Scotland

^3^Department of Plastic Surgery, St Thomas’ Hospital, Guys and St Thomas’ Trust, London

**Corresponding author.** Sean C. Glossop, Cardiff University School of Medicine,

Cardiff University Heath Park Campus,

Cardiff, CF14 4YS **ORCID ID**; 0000-0001-8495-0860

**Supplementary Materials - Index**

| **Supplementary Figures and Tables** |  |
| --- | --- |
| Table S1 - A breakdown of all studies used in the review | *pages 2-7* |
|  |  |
| **References** | *pages 8-9* |
|  |  |

**Supplementary Figures and Tables**

**Table S1 – A breakdown of all studies used in the review**

| Study & Year | Design | Sample Size | Data Collection Method | Intervention | Findings |
| --- | --- | --- | --- | --- | --- |
| Lee et al (2016)^1^ | Cross-sectional study | 328 medical students. | Survey to medical schools and a separate survey to medical students. |  | 65% of students felt prepared for surgical foundation jobs which is significantly less compared with the 88% of students who felt prepared for medical foundation placements. 33% of the students felt their experience of surgical science teaching in medical school was adequate with 80% being confident in surgical scrubbing, 60% in local anaesthetics and 45% in wound care. |
| Davis et al (2014)^2^ | National review questionnaire | 23 medical schools in the UK. | Questionnaire sent to 32 medical schools with presidents of each surgical society selected as responders to represent their corresponding medical school. |  | 72.8% of medical schools taught “gowning and gloving” whilst  29.4% taught “handling instruments”. However “knot tying” and “suturing techniques” were taught by 17.4% and 24.7% of medical schools respectively with these skills being taught statistically more by RCS surgical societies (65.4% and 64.5%, p<0.01). |
| Rufai et al (2015)^3^ | Survey | 705 medical students (from 16 medical schools). | Survey sent to 17 medical schools to be completed by students. |  | 86% of students surveyed reported being taught suturing in medical school and of this 86%, 13.5% thought that this training was adequate. Also, the results for all markers of competency tested were significantly lower than the expected outcome of 100% compliance with GMC standards (p<0.001). |
| Lee et al (2020)^4^ | Survey | 29 medical students. | Pre and post intervention self assessment and instructor assessment forms. | Surgical skills day aiming to provide additional training on basic wound closure skills which taught and assessed seven skills mandated by the GMC for foundation doctors. | Mean improvement of 3.92 points (±0.40) (P<0.001) before and after instruction for suturing (marked for each student, by an instructor, using a binary system where 0 and 1 represent incompetency and competency respectively). 100% of participants agreed that increased practical surgical skills should be added to the undergraduate curriculum. |
| Hakim et al (2019)^5^ | Survey | 107 medical students. | Anonymised feedback form completed after the surgical skill session. | An annual surgical skills day, over the course of three years which provided workshops on a variety of practical surgical skills. The instructors running the workshops ranged from surgeons at trainee to consultant level. | Mean student satisfaction was greater than 7/10 for all stations. 79% of students said the surgical skills day made them more likely to follow a career in surgery with student comments (71.5%) at the 2018 event reporting positive interactions with the surgeon instructors. 97% said they wanted “practical surgical skills” as part of the medical school curriculum. |
| Kuo et al (2022)^6^ | Survey | 58 phase one medical students. | Anonymised feedback before the course, after each of the sessions and then upon completion. | An in-person surgical skills course with five sessions guided by the GMC’s ‘Outcomes for graduates’ and led by tutors with surgical experience. | Confidence levels for all GMC mandated skills were increased before and after the intervention, at statistical significance and 63.3% of participants felt that the course had a large impact on their confidence in carrying out basic surgical skills. Additionally, confidence levels in how prepared participants felt, within their skill sets, for future placements and work increased postcourse. |
| Spiers et al (2018)^7^ | Questionnaire | 22 responses from medical students. | Questionnaires given before and after the surgical skills day. | Plastic surgery skills day consisting of lectures followed by interactive surgical skills workshops in suturing, tendon repair and local flap design. | Self-assessed feedback showed mean improvement for basic and more advanced surgical skills with all reported outcomes seeing positive changes in the confidence levels of participants in a variety of surgical skills after the intervention. |
| Spiers et al (2019)^8^ | Questionnaire | 35 responses from medical students. | Questionnaires given before and after the surgical conference. | One day course comprising lectures followed by surgical skills workshops, focusing on the specialty of ENT. The designated taught skill chosen was tracheostomy. | The post-course questionnaires showed an increase in positive perceptions of ENT and a reduction in negative perceptions with perceptions of ENT being improved by 80% (p<0.01). There was a statistically significant increase in participants' self-reported confidence surrounding tracheostomy with a 3.17 increase (p<0.0001) seen in participants’ confidence of performing the procedure post-course. |
| George et al (2017)^9^ | Likert-style questionnaire | 80 medical students. | Questionnaire given before and after the course. | A surgical skills workshop focused on cardiothoracic surgery which included several workshops: guided dissections of hearts, surgical skills practice, operative videos with commentary and a careers seminar. | Statistically significant increase (p<0.001) in mean Likert scores after the workshop compared to before the workshop which represented a 28% increase in interest in cardiothoracic surgery as a result of the intervention. |
| Mughal et al (2015)^10^ | Questionnaire | 46 students completed pre intervention questionnaire, 60 completed post-intervention. | Pre and post intervention questionnaire and 26 item multiple choice question quiz. | A national surgical workshop that focused on increasing knowledge and skill in the early recognition and management of acutely unwell surgical patients.  The workshop included: high-fidelity simulations, lectures, case demonstrations, discussions and a surgical skills demonstration. | 8 confidence and 4 self confidence statements - statistically significant improvement in all statements on the questionnaires before and after (p<0.0001). The MCQ scores immediately following the intervention were statistically improved compared with prior to the workshop and this improvement was sustained eight weeks following the workshop. |
| Down et al (2020)^11^ | Likert-style questionnaire | 14 medical students. | Pre and post intervention questionnaire. | A combined near peer and faculty-led surgical skills course where students were taught basic skills and then advanced tasks were designed to consolidate the student’s knowledge in clinical practice. | The self-reported mean confidence improved significantly in the post intervention questionnaire with an improved confidence in all skills by +1.254 (p<0.0001). Participants reported improved confidence in basic skills with each improvement being significant. Handling instruments, interrupted suturing, continuous suturing, subcuticular suturing and knot tying improved by +0.65, +0.9, +1.45, +1.33 and +1.22 respectively. As well as this, reported confidence was improved for advanced skills post intervention. |
| Chidambaram et al (2019)^12^ | Randomised controlled trial | 21  medical students in the intervention group and 17 medical students in the control group. | Performance of the control and intervention group was compared using a validated scoring tool on a porcine laparoscopic cholecystectomy model. An independent assessor rated the recordings. | Both the intervention and control group received a ten minute introduction to laparoscopic equipment as well as a fifteen minute tutorial on laparoscopic cholecystectomies before the intervention.  Intervention group- laparoscopic cholecystectomy phase 3 module on the touch surgery (TS) training application for a limit of fifteen minutes.  Control group- fifteen minutes studying written information breaking down the intraoperative steps of laparoscopic cholecystectomies. | The overall mean cognitive performance score was greater for the intervention group (41.9) than the control group (24.7). However, this difference wasn’t statistically significant (P>0.05). |
| Bennett et al (2018)^13^ | Likert-style questionnaire | 70 students including: 59 medical, 2 dental and 9 physician associate students. | Pre and post intervention questionnaire. | A half day surgical skills course in a peer assisted learning (PAL) environment focusing on eight domains in surgery: WHO surgical safety checklist, scrubbing in, gowning/gloving, knot tying, interrupted sutures, continuous sutures, vertical mattress sutures and local anaesthesia. | There was an increase in self-reported mean confidence scores post intervention across all the eight domains covered in the course. Of the eight domains, half saw increases in confidence which were significant: knot tying (+5.53), interrupted sutures (+5.89), continuous sutures (+6.53) and vertical mattress sutures (+6.46, p<0.05). As well as this, 80% of participants reported that the course had developed their interest in pursuing a career in surgery. |
| Preece et al (2015)^14^ | Questionnaire | 35 second and third year medical students. | Pre and post intervention questionnaire. Also, a ten minute suturing assessment, the results of which were analysed by a blinded investigator. | Two three hour peer assisted learning suturing workshops covering the concepts of wound closure, basic knot tying and suturing techniques. The workshop involved a presentation followed by practical experience of the skills covered. | The results from the suturing assessment showed that following teaching, the mean number of completed sutures increased significantly from pre-teaching to post-teaching (p<0.001). All of the participants felt that the workshop had improved their suturing skills and confidence with 87% of them feeling that the workshop had increased their interest in pursuing a career in surgery. |
| Saleh et al (2013)^15^ | Qualitative |  | Medical students recounting their experiences as a session organiser, peer tutor and pre-clinical student. | Experience of peer assisted learning workshop to teach basic surgical skills as a session organiser, peer tutor and pre-clinical student. | Session organiser- this experience, whilst considered “challenging” enabled the students to develop “organisational and leadership skills”.  Peer tutor- this role involved “demonstrating basic suturing”, allowing the tutor to “positively reinforce” their “own abilities” whilst improving their “presentation skills” and resulting in a “strengthened relationship with peers”.  Pre-clinical student- the chance to develop basic suturing skills in a peer assisted learning environment which was “informal” helped the student to “really concentrate on learning the skills”. |
| Sutton et al (2014)^16^ | Survey | 482 final year medical students from 20 medical schools throughout England and Wales. | A survey designed to collect students’ perceptions on careers in surgery as well as their early experiences in surgery. |  | The two most common factors discouraging medical students from pursuing a career in surgery were clinical placements whilst at medical school (43%) and work experience (35%). 91% of respondents had work experience prior to medical school, with the greatest satisfaction reported in the 21% who undertook a surgical placement. Students reported the least exposure to oral and maxillofacial surgery, cardiothoracic surgery and plastic surgery within the curriculum. |
| Rouhani et al (2017)^17^ | Survey | 137 medical students. | A web-based surgery was distributed via email to medical schools. |  | 73% of respondents (100) stated they were interested in a career in surgery with 62 students considering this their first choice specialty. Some reasons respondents weren’t considering a career in surgery included surgeon’s attitudes, job competitions and limited opportunities for learning in theatre. |
| Ologunde et al (2015)^18^ | Cross sectional survey | 60 undergraduate and postgraduate students | An anonymous ten question survey |  | Surgical skills workshops (21.9%), conferences (21.1%) and career talks (16.4%) were chosen by respondents as the most useful career-guiding events organised by surgical societies. Also, 62.3% of respondents showed that the society had increased their knowledge about the different surgical societies. |
| Hamaoui et al (2013)^19^ | Likert-style questionnaire | 47 third and fourth year medical students. | Pre and post intervention questionnaire. | A surgical skills course based on the Royal College of Surgeons’ Basic Surgical Skills Course which covers basic skills such as knot tying as well as the more complex insertion of a chest drain. | There was a significant increase in participants reported confidence in all four skills taught post intervention (p<0.01) with 80% of participants feeling better prepared for their surgical rotations as well as other surgical specialty rotations like emergency medicine and obstetrics and gynaecology. Additionally, post intervention, 70% of participants reported that attending such a course could influence future students in their career decisions. |

**References**

1. Lee MJ, Drake TM, Malik TAM, O’Connor T, Chebbout R, Daoub A, et al. Has the Bachelor of Surgery Left Medical School?—A National Undergraduate Assessment. J. Surg. Educ. 2016;73(4):655-659.

2. Davis CR, Toll EC, Bates AS, Cole MD, Smith FCT. Surgical and procedural skills training at medical school – a national review. International Journal of Surgery. 2014;12(8):877-882.

3. Rufai SR, Holland LC, Dimovska EO, Bing Chuo C, Tilley S, Ellis H. A National Survey of Undergraduate Suture and Local Anesthetic Training in the United Kingdom. J. Surg. Educ. 2016;73(2):181-184.

4. Lee KS, Priest S, Wellington JJ, Owoso T, Osei Atiemo L, Mardanpour A, et al. Surgical Skills Day: Bridging the Gap. Cureus. 2020;12(5):e8131.

5. Hakim MA, Dominguez ED, Priest S, Lee KS, Mardanpour A, Tandle S, et al. Surgical Skills Workshops Should Be a Part of the United Kingdom Undergraduate Medical Curriculum. Cureus. 2019;11(5):e4642.

6. Kuo L, Salloum NL, Kennard B, Robb J, Vickerton P. Impact of an in-person small group surgical skills course for preclinical medical students in an era of increased e-learning. Surgery Open Science. 2022;10:148-155.

7. Spiers HVM, Zargaran A, Murtaza AN, Thomas A, Turki MAA, Ali F. Enhancing Medical Curricula: The Role of a 1-Day Plastic Surgery Course as an Educational Adjunct for Medical Students. J. Surg. Educ. 2018;75(1):116-121.

8. Spiers H, Enayati H, Moussa R, Zargaran A, Thomas A, Murtaza A, et al. Augmenting ENT surgery outside the medical school curriculum: the role of a 1-day otolaryngology course. The Journal of Laryngology & Otology. 2019;133(4):269-274.

9. George J, Combellack T, Lopez-Marco A, Aslam U, Ahmed Y, Nanjaiah P, et al. Winning Hearts and Minds: Inspiring Medical Students into Cardiothoracic Surgery Through Highly Interactive Workshops. J. Surg. Educ. 2017;74(2):372-376.

10. Mughal Z, Isherwood JD, Boam TD, Knight SR, Yeung JMC. Development, Evaluation, and Delivery of an Innovative National Undergraduate Surgical Workshop: Recognition and Management of the Acutely Unwell Surgical Patient. Teach. Learn. Med. 2015;27(1):85-90.

11. Down B, Morris S, Kulkarni S, Mohiuddin K. Effectiveness of a multi-session combined near-peer and faculty-led surgical skills course on self-perceived ability to perform basic surgical skills. Annals of Medicine and Surgery. 2020;57:153-156.

12. Chidambaram S, Erridge S, Leff D, Purkayastha S. A Randomized Controlled Trial of Skills Transfer: From Touch Surgery to Laparoscopic Cholecystectomy. J. Surg. Res. 2019;234:217-223.

13. Bennett SR, Morris SR, Mirza S. Medical Students Teaching Medical Students Surgical Skills: The Benefits of Peer-Assisted Learning. J. Surg. Educ. 2018;75(6):1471-1474.

14. Preece R, Dickinson EC, Sherif M, Ibrahim Y, Ninan AS, Aildasani L, et al. Peer-assisted teaching of basic surgical skills. Med. Educ. Online. 2015;20:27579.

15. Saleh M, Sinha Y, Weinberg D. Using peer-assisted learning to teach basic surgical skills: medical students' experiences. Med. Educ. Online. 2013;18:21065.

16. Sutton PA, Mason J, Vimalachandran D, McNally S. Attitudes, Motivators, and Barriers to a Career in Surgery: A National Study of UK Undergraduate Medical Students. J. Surg. Educ. 2014;71(5):662-667.

17. Rouhani M, Gelder C, Selwyn-Gotha J, Rufai S. Which factors influence the pursuit of a career in surgery? A national undergraduate survey. British Journal of Healthcare Management. 2017;23(12):581-587.

18. Ologunde R, Rufai SR, Lee AHY. Inspiring Tomorrow’s Surgeons: The Benefits of Student Surgical Society Membership☆?>. J. Surg. Educ. 2015;72(1):104-107.

19. Hamaoui K, Sadideen H, Saadeddin M, Onida S, Hoey AW, Rees J. Is it time for integration of surgical skills simulation into the United Kingdom undergraduate medical curriculum? A perspective from King's College London School of Medicine. J Educ Eval Health Prof. 2013;10:10.
